# Novel RNA biomarkers of prostate cancer revealed by RNA-seq analysis of formalin-fixed samples obtained from Russian patients

**DOI:** 10.18632/oncotarget.16518

**Published:** 2017-03-23

**Authors:** Anastasia S. Nikitina, Elena I. Sharova, Svetlana A. Danilenko, Tatiana B. Butusova, Alexandr O. Vasiliev, Alexandr V. Govorov, Elena A. Prilepskaya, Dmitry Y. Pushkar, Elena S. Kostryukova

**Affiliations:** ^1^ Federal Research and Clinical Center of Physical-Chemical Medicine, Moscow, Russia; ^2^ Moscow Institute of Physics and Technology, Dolgoprudnyi, Russia; ^3^ Department of Urology, Moscow State Medical Stomatological University, Moscow, Russia

**Keywords:** prostate cancer, benign prostatic hyperplasia, RNA biomarkers, RNA-Seq, FFPE

## Abstract

Due to heterogeneous multifocal nature of prostate cancer (PCa), there is currently a lack of biomarkers that stably distinguish it from benign prostatic hyperplasia (BPH), predict clinical outcome and guide the choice of optimal treatment. In this study RNA-seq analysis was applied to formalin-fixed paraffin-embedded (FFPE) tumor and matched normal tissue samples collected from Russian patients with PCa and BPH. We identified 3384 genes differentially expressed (DE) (FDR < 0.05) between tumor tissue of PCa patients and adjacent normal tissue as well as both tissue types from BPH patients. Overexpression of four of the discovered genes (ANKRD34B, NEK5, KCNG3, and PTPRT) was validated by RT-qPCR. Furthermore, the enrichment analysis of overrepresented microRNA and transcription factor (TF) recognition sites within DE genes revealed common regulatory elements of which 13 microRNAs and 53 TFs were thus linked to PCa for the first time. Moreover, 8 of these TFs (FOXJ2, GATA6, NFE2L1, NFIL3, PRRX2, TEF, EBF2 and ZBTB18) were found to be differentially expressed in this study making them not only candidate biomarkers of prostate cancer but also potential therapeutic targets.

## INTRODUCTION

Prostate cancer (PCa) is the most commonly diagnosed and third-leading cause of cancer-related death among men in developed countries [1]. Such highly prevalent malignancy is obviously subjected to numerous studies including those aimed at finding its specific biomarkers. However nowadays there is still a lack of reliable prognostic biomarkers as well as the diagnostic ones for consistently distinguishing prostate cancer from benign prostatic hyperplasia (BPH).

The perspective way of searching for such biomarkers is to conduct the analysis of gene expression in prostate tissue by means of RNA sequencing (RNA-Seq). Unlike microarrays and real-time PCR this approach allows the *de novo* detection of both protein coding and non-coding RNAs. The latter is of a particular interest in light of growing number of studies indicating that non-coding RNAs, antisense RNAs and pseudogenes play a significant part in PCa development and progression [2–4]. Identification of differentially expressed genes encoding these and other types of transcripts within the prostate tissue may reveal expression signatures associated with clinical manifestations of PCa.

The aim of this study was to analyze the expression profiles of matched tumor and adjacent normal tissue samples obtained from patients with PCa and BPH from Russian Federation. We also assessed the effectiveness of RNA-Seq approach applied to formalin-fixed, paraffin embedded (FFPE) tissue samples and evaluated the consistency of the results obtained with morphological features of the samples and clinical characteristics of the patients.

## RESULTS AND DISCUSSION

### Analysis of composition and depth of the transcriptional profiles obtained

The first stage of the study included RNA samples extracted from thin FFPE sections of prostate tissue obtained from 15 patients with PCa and 2 patients with BPH. For 15 patients there were matched samples, i.e. RNA was isolated from both tumorous tissue and histologically unchanged adjacent tissue, resulting in thirty samples total. For two patients only “tumor” samples were used (Table 1).

**Table 1:** Clinical characteristics of the patients

Patient ID	Diagnosis	Age at operation	PSA, ng/ml	Gleason score			Sample ID		Final set
				Sum	Primary	Secondary	Pathology	Normal	
**A50_002**	BPH	67	1.5	-	-	-	**BP1**	**BN1**	ν
**A50_004**	BPH	68	9.6	-	-	-	**BP2**	**-**	ν
**A50_006**	PCa	64	17	7	4	3	**BP3**	**BN3**	ν
**P50_001**	PCa	60	10	7	3	4	**CP1**	**CN1**	ν
**P50_002**	PCa	55	8.6	7	4	3	**CP2**	**-**	ν
**P50_004**	PCa	55	19	8	4	4	**CP3**	**CN3**	ν
**P50_007**	PCa	59	5	6	3	3	**CP5**	**CN5**	
**P50_008**	PCa	69	15	5	3	2	**CP6**	**CN6**	
**P50_009**	PCa	57	16.6	9	4	5	**CP7**	**CN7**	
**P50_010**	PCa	69	5.3	7	3	4	**CP8**	**CN8**	ν
**P50_011**	PCa	67	5.6	7	4	3	**CP9**	**CN9**	
**P50_013**	PCa	56	15	7	4	3	**CP10**	**CN10**	
**P50_015**	PCa	48	7.8	6	3	3	**CP11**	**CN11**	ν
**P50_016**	PCa	67	6.6	5	3	2	**CP12**	**CN12**	ν
**P50_019**	PCa	73	3.9	7	4	3	**CP13**	**CN13**	ν
**P50_020**	PCa	50	12	7	3	4	**CP14**	**CN14**	ν
**P50_022**	PCa	67	6.1	6	3	3	**CP15**	**CN15**	ν

Samples that were included in the final sample set for differential expression analysis are marked.

For each RNA sample transcriptome libraries were constructed and sequenced using the semiconductor sequencing technology. The obtained reads were filtered by quality and mapped to human genome version hg19. Reads corresponding to ribosomal RNAs did not present any interest for further analysis and therefore were excluded. Among other reads mapped to annotated sequences of the genome were the ones originated from protein-coding transcripts, long non-coding RNAs, small nucleolar RNAs, antisense RNAs, pseudogenes and other types of non-coding transcripts (Table 2). On average the transcripts of ~18000 genes were detected for each sample. According to EMBL-EBI Expression Atlas [5], the number of genes whose transcripts are found in prostate tissue varies from 11000 to 18000 based on the results of The FANTOM5 project» [6] and «GTEx» [7]. Thus the RNA-Seq analysis of RNA samples isolated from thin paraffin sections of prostate tissue provided the expression profiles reflecting the substantial part of the transcriptional activity of this tissue.

**Table 2:** Composition of transcriptional profiles

	Fraction of genes (range), %	Fraction of genes (mean), %	Fraction of reads (range), %	Fraction of reads (mean), %
**Protein-coding transcripts**	73–84	76	51–85	65
**Long non-coding RNAs**	3–6	5	5–9	6
**Small nucleolar RNAs**	1–2	1	1–11	6
**Small nuclear RNAs**	1–2	2	1–12	7
**Antisence RNAs**	2–5	4	2–5	3
**Pseudogenes**	4–7	6	0.3–0.7	0.5
**Other transcripts**	4–7	5	5–22	12

Fraction of genes encoding a particular transcript of all detected genes for each sample and fraction of reads mapped to such genes of all reads were calculated. Corresponding range and mean values are indicated.

### Sample clustering and the final sample set selection

**Figure 1:**
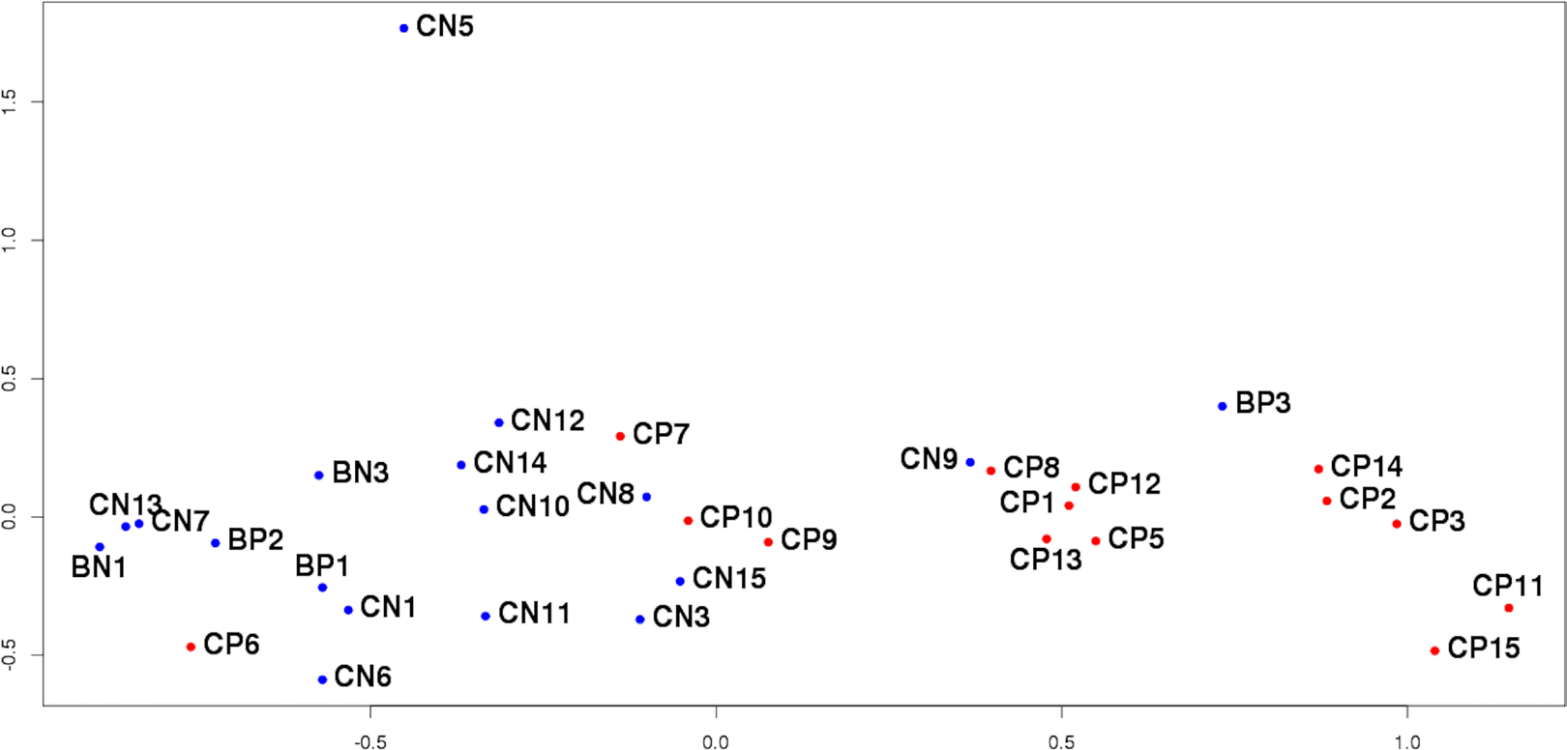
MDS-plot of all sequenced samples The distance between the dots represents the similarity of corresponding samples’ transcriptional profiles.

In order to assess the uniformity of the sample set multidimensional scaling (MDS) was used to cluster samples based on their expression profiles (Figure 1). It is clear that such clusterization successfully separates “tumor” samples from “normal” ones, although there are still several outliers not fitting in the whole picture. Corresponding thin FFPE sections were subjected to the second pathomorphological study. The greatest difference from other samples appears to be exhibited by expression profile of the sample CN5 obtained from “normal’ tissue of the patient with PCa. The second histological analysis revealed that the significant part of the “normal” prostate tissue on the FFPE section was taken up by the seminal vesicle, RNA-composition of which could naturally be found completely different. Samples CN5/CP5 were excluded from further analysis.

Next sample that draws attention is CP6 from tumor tissue clustered with “normal” samples. Its FFPE section turned out to contain very small fraction of tumor tissue compared to histologically unchanged adjacent tissue, and consequently during the process of RNA extraction from that FFPE section high proportion of “normal” tissue could have been taken. The same applies to sample CP7. Samples CP6, CP7 and their counterparts were excluded from further analysis.

In the case of sample CP10, the secondary histological study of the corresponding FFPE-section revealed a piece of normal tissue of a significant area within the tumor. A similar situation was observed for samples CP9/CN9 located on the border of tumor and normal groups on Figure 1. Here it is worth quoting the conclusion of the second pathomorphological report: “Tumor grows infiltratively, i.e. complexes of tumor cells not only form isolated bundle but also penetrate healthy tissue. As a result, the site initially marked out as tumor also included healthy tissue infiltrated by tumor complexes. It is impossible to provide a clear distinction between tumor and normal areas for this section”. In both described cases the presence of complicated sites resulted in the incorrect marking of the sections, which in turn prevented from obtaining strictly tumorous and normal RNA pools. Samples CN9/CP9 and CN10/CP10 were excluded from further analysis.

The situation was different for sample BP3, obtained from patient with BPH according to accompanying documents. However, unlike other “adenomatous” samples (BN1, BP1, BP2), expression profile of BP3 resembles those of “tumor” samples. As a result of thorough inspection an error was found in patient's documents and the diagnosis was in fact PCa. Interestingly, it was not possible to discover this mistake at any previous stage of the research and only the comparison of the expression profiles pointed out the similarity of BP3 to “tumor” samples. This sample was not excluded from analysis but was added to tumor comparison group.

Thus, the obtained transcriptional profiles reflect the specific features of the samples and patients’ clinical characteristics, which is essential when searching for biomarkers capable of distinguishing different phenotypes. These results confirm the need to examine the sample groups for internal homogeneity since the outliers may distort the picture and lower the quality of the research. The results obtained also demonstrate high efficiency of clusterization based on the expression profiles to identify samples not suitable for further analysis.

### Differential expression analysis

**Figure 2:**
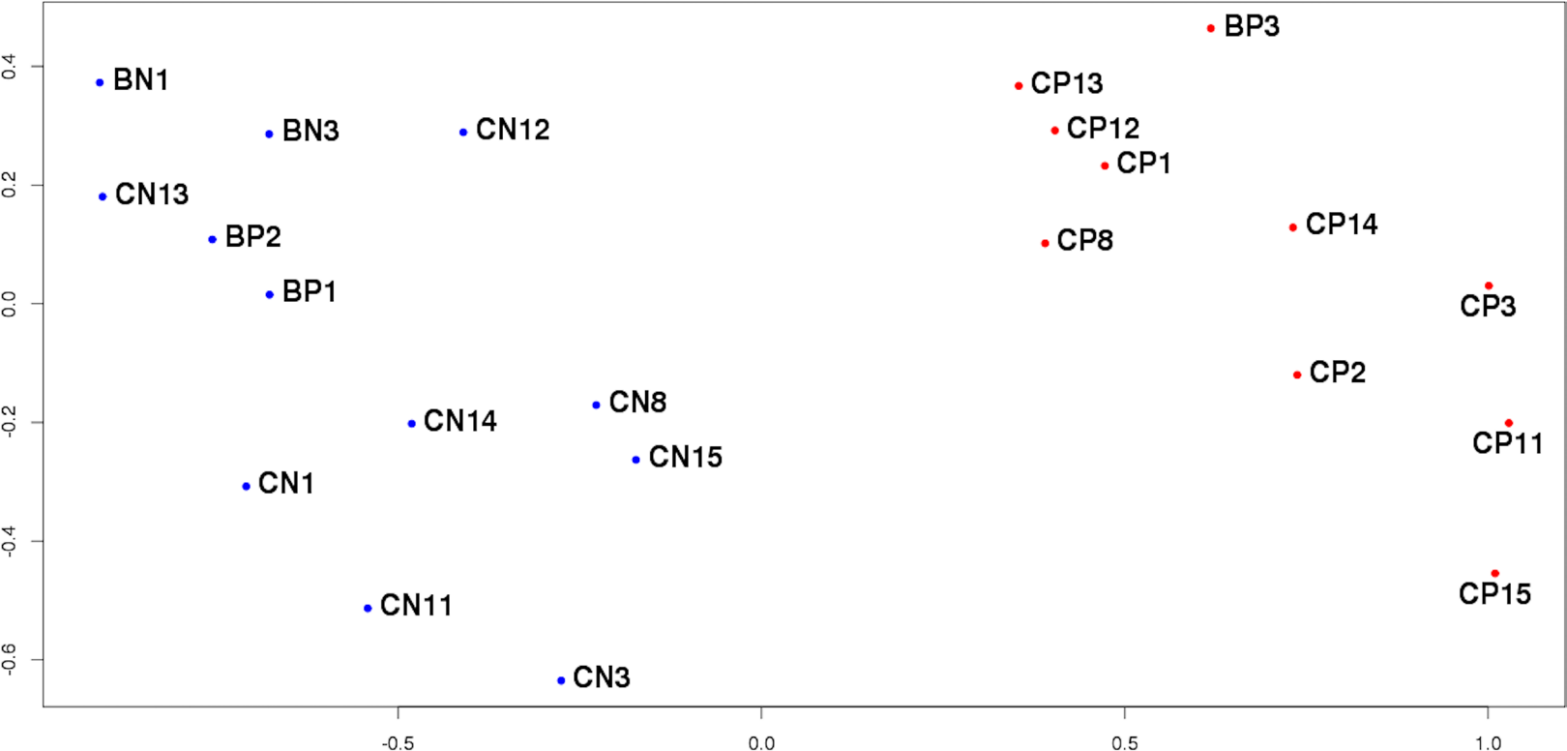
MDS-plot of the final sample set The distance between the dots represents the similarity of corresponding samples’ transcriptional profiles.

After removing the outliers identified above the final sampling consisted of 22 samples divided into control and tumor groups (Table 1). Because of the current lack of biomarkers for differential diagnostics of PCa and BPH, the control group of the study aimed at identifying potential markers of PCa should include samples obtained from patients with BPH [8]. For this reason, the control group in this investigation contained 12 samples, 9 of which corresponded to histologically unchanged adjacent to tumor tissue of patients with PCa (BN3, CN1, CN3, CN8, CN11, CN12, CN13, CN14, CN15), 1 – to histologically unchanged adjacent to tumor tissue of patients with BPH (BN1), and 2 – to hyperplastic tissue of patients with BPH (BP1, BP2). Tumor group comprised 10 samples of tumorous tissue of patients with PCa (BP3, CP1, CP2, CP3, CP8, CP11, CP12, CP13, CP14, CP15). Based on the sequencing data of these 22 samples the analysis of differential expression was conducted in order to discover potential biomarkers of PCa. The MDS-plot reconstructed only for these samples (Figure 2) shows clear separation of the comparison groups. Moreover, it is worth noticing that the control group splits into two clusters: one of them contains samples from BPH patients and the other one – from PCa patients.

The differential expression (DE) analysis resulted in the identification of 3384 genes (Supplemtary Table 1), whose expression differed between specified comparison groups with the statistical significance level set at FDR < 0.05. These genes are diverse in the types of transcripts they encode (Table 3). Interestingly, 371 (11%) of them correspond to non-coding transcripts, confirming the significant role these RNAs play in carcinogenesis [9, 10]. Furthermore, a high portion (2,7%) of DE small nucleolar RNAs is also worth noting with most of them being upregulated in tumor tissue. These results validate the involvement of this gene type in the process of PCa progression, which had been recently proposed [11, 12].

**Table 3:** Number of differentially expressed genes encoding different types of transcripts

	DE genes	%	Upregulated	%	Downregulated	%
**Protein-coding transcripts**	3013	89	1246	36.8	1767	5.2
**Long non-coding RNAs**	91	2.7	53	1.6	38	1.1
**Small nucleolar RNAs**	92	2.7	77	2.3	15	0.4
**Small nuclear RNAs**	1	0.03	1	0.03	0	0
**Antisence RNAs**	52	1.5	27	0.8	25	0.7
**Pseudogenes**	51	1.5	30	0.9	21	0.6
**Other transcripts**	84	2.5	56	1.7	28	0.8
**Total**	3384	100	1490	44	1894	56

Corresponding numbers for upregulated and downregulated in tumor tissue genes are indicated.

The list of DE genes identified in this study included many already known biomarkers of PCa, according to the literature. First of all, prostate cancer antigen 3 (PCA3) – long non-coding RNA, associated with PCa for the first time by its high expression level in tumorous tissue [13], – were also significantly overexpressed in “tumor” samples in this study (logFC = 4.6, FDR = 1.3e-12). Today PCA3 is being introduced into clinical practice as diagnostic marker of PCa detectable in urine, characterized by low false-negative rate and higher specificity than prostate specific antigen (PSA) [14]. It is worth noting that KLK3 gene, encoding PSA protein, did not appear on the list of statistically significant DE genes, which agrees well with its low specificity with respect to BPH [15]. Differential expression of other known candidate biomarkers was detected in this study, including alpha-methylacyl-CoA racemase (AMACR) – mitochondrial beta-oxidase [16], – and genes HOXC6, TDRD1 and DLX, that form recently suggested three-gene panel, developed for early diagnostics of aggressive PCa (Gleason score 7 and higher) based on detection of these genes’ RNA in urine and measuring the level of serum PSA [17]. One more gene Ankyrin Repeat Domain 34B (ANKRD34B or DP58) less known as PCa biomarker but shown to be differentially expressed in prostate tumor/normal tissue comparison [18] was also found to be significantly overexpressed in tumor tissue in this study. Observed differential expression of these genes proves their value as PCa biomarkers and reflects the quality of the conducted research.

Besides already known candidate markers we managed to detect changes in mRNA levels of genes whose disrupted expression had not been previously associated with prostate cancer. Three of these genes are NEK5, KCNG3, and PTPRT. Expression pattern of the first one, NIMA related kinase 5 (NEK5), is poorly characterized at the moment. It belongs to the family of NEK kinases and is known to participate in cell cycle progression [19] and, as has recently been shown, in the regulation of mitochondrial mediated cell death [20]. The next two genes: KCNG36, encoding the subunit of potassium voltage-gated channel, and PTPRT, encoding tyrosine phosphatase, had not been associated with PCa previously. However, they had been linked to other malignant neoplasms in the context of corresponding CpG hypermethylation [21, 22].

### Comparison to TCGA data

Next, the list of DE genes obtained for samples from Russian Federation was compared to the results of analysis of a wider sample set. As such, The Cancer Genome Atlas (TCGA) [23] data was used, specifically the results of RNA sequencing of 90 matched prostate tissue samples of 45 patients with PCa. Raw sequencing data was downloaded and analyzed using the same pipeline as Russian set. Evaluation of the TCGA sampling uniformity by MDS revealed several outliers, specifically 7 samples which corresponded to normal tissue. Since there were no possibility to conduct the second pathomorphological analysis of original tissues, we performed the differential expression analysis between these 7 samples and other TCGA samples derived from normal tissue. The transcriptional profiles of these 7 samples were found to match those of seminal vesicles rather than prostate tissue. The same case has been described above for Russian set. This problem might be common when working with tissues from patients with PCa, which supports once more the need to examine the uniformity of sample groups. These 7 samples and their matched ones were excluded from the further analysis which reduced the number of samples to 76 (38 in each comparison group).

**Figure 3:**
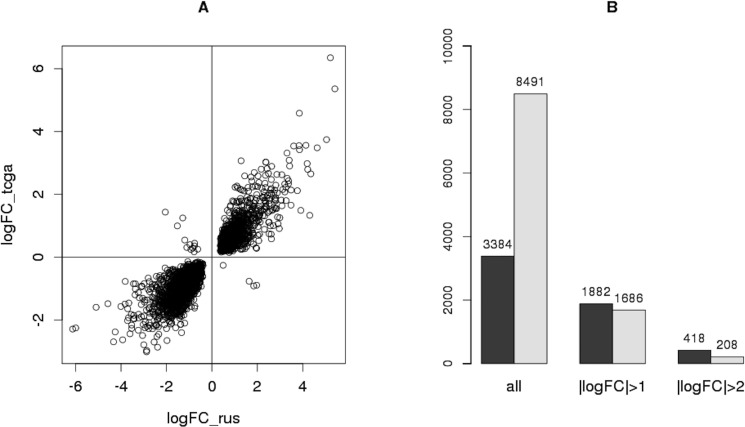
Comparison of the results obtained with TCGA data (**A**) Scatter-plot showing the direction and value of the expression change of every gene differentially expressed both in Russian sample set (logFC_rus) and in TCGA data (logFC_tcga). (**B**) Number of all DE genes in each sample set (dark - Russian, light – TCGA) whose expression change satisfies a certain condition.

The differential expression analysis led to discovery of 8491 DE genes (FDR < 0.05), 2631 of which were found to be common for TCGA and Russian Federation data obtained in this study (Supplementary Table 2). Moreover, the expression of 2615 of these 2631 common genes changed in the same direction in both sample sets (Figure 3A), and Pearson correlation of the corresponding logFC (log2(expression fold change)) values was *r* = 0.92. Such coalignment and high correlation of expression levels of these genes in two independent groups of samples obtained from patients with PCa confirm the validity of the hypothesis of these genes’ possible association with this disease.

Furthermore, the number of genes with particular logFC were compared between these sample sets (Figure 3B). Despite the fact that the number of all DE genes for TCGA data (8491) greatly exceeds that for Russian Federation sampling (3384), most of them have |logFC| < 1, meaning that the expression of these genes changed by less than a factor of 2. Conversely, the number of genes with |logFC| > 1 and |logFC| > 2 is higher for the sample set obtained in this study (Figure 3C). This effect might be explained by different sizes of sample sets (76 samples in TCGA and 22 in Russian data). The type of starting material is worth taking into account as well: whereas thin paraffin sections were used in this study, the TCGA data were obtained using samples of frozen tissue, the material more challenging for pathomorphological assessment especially in the case of such multifocal cancer as PCa.

Thus, the results of differential expression analysis conducted in this study are in high concordance with the data independently obtained for the wider sample set. Moreover, dealing with a small but uniform sample set with carefully selected samples allows to discover more genes with significant expression change during PCa, which is the key stage in searching for potential RNA-markers.

### Validation of RNA-Seq results

Results obtained in this study by RNA-Seq were verified by RT-qPCR on a wider sample set, which contained 12 samples from the original sequenced set and 23 new samples. Overall validation was performed on 35 samples (Supplementary Table 3) from 17 patients with PCa and 3 patients with BPH. As in the case of differential expression analysis, the control group included samples from histologically unchanged adjacent to tumor tissue of patients with PCa and all of the samples from BPH patients.

One of the main criteria of the search for potential RNA-markers is their ease of detection by a clinically available method like RT-qPCR. For this reason, only genes upregulated in tumor tissue with significant expression change of logFC > 3 were considered when choosing candidate biomarkers. The level of statistical significance was also selected to be stricter (FDR < 0.01). Finally, despite discovering several non-coding RNAs which meet all these criteria and are of interest for further research, only protein-coding transcripts having long introns were taken into consideration in order to facilitate cDNA analysis by RT-qPCR. As a result of such gradual reduction of the DE gene list, 4 genes were chosen for validation by real-time PCR: ANKRD34B, NEK5, KCNG3, and PTPRT. Overexpression of the last three of them in tumor prostate tissue was demonstrated in this study for the first time.

The results of RNA-Seq showed expression of the first gene ANKRD34B in tumorous prostate tissue (9/10 samples) and nearly no expression in control group (2/12 samples). These results were validated by qPCR: the expression was not detected in 22 of 23 normal samples, whereas it was observed in 8 of 12 tumor samples. The subsequent application of the Fisher's exact test indicated the level of statistical significance of these results to be *p* = 2.0e-04.

**Figure 4:**
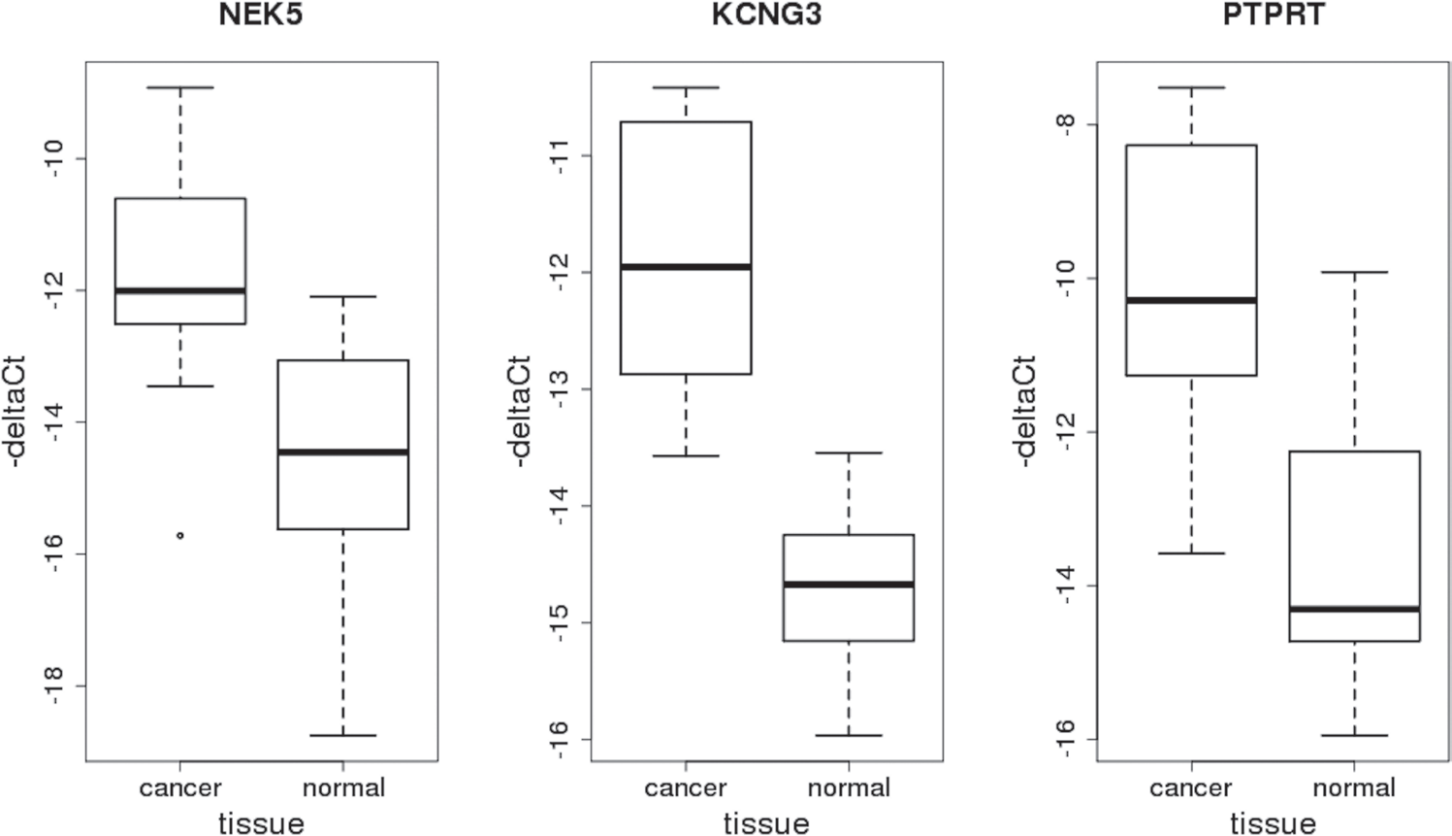
Box-plots representing relative expression levels of genes NEK5, KCNG3 and PTPRT obtained by RT-qPCR

Expression of the other three genes on RNA level was detected by RT-qPCR in both comparison groups. In concordance with RNA-Seq results, it was found to be higher in tumor samples (Figure 4). The corresponding deltaCt values were compared with the application of non-parametric Wilcoxon test which showed the levels of statistical significance to be p_NEK5_ = 5.7e-03, p_KCNG3_ = 5.3e-05 and p_PTPRT_ = 3.0e-04.

Additionally, it is worth noting that according to the data from Human Protein Atlas obtained by immunohistochemistry [24], protein products of three of four investigated here genes ANKRD34B [25], NEK5 [26] and PTPRT [27] were elevated in tumor prostate tissue compared to histologically unchanged tissue. There was no available data for KCNG3.

Thus, overexpression of genes ANKRD34B, NEK5, KCNG3 и PTPRT in samples of tumorous prostate tissue compared to control can serve as the new easy detectable marker of PCa. Further research should be aimed at developing diagnostic panels based on these and known potential biomarkers mentioned above.

### Identification of novel regulatory elements

After obtaining the list of 3384 DE genes it is interesting to determine gene subgroups with common mechanisms influencing their transcript expression. The main elements carrying out such regulation are transcription factors (TF) affecting transcription of many genes, and microRNAs, capable of regulating mRNA degradation or translation. To discover these elements, the obtained list of 3384 DE genes was divided into 2 groups: genes with lower expression level in tumor tissue compared to control (logFC < 0, 1894 genes) and genes with higher expression level (logFC > 0, 1490 genes). Genes without Entrez ID were excluded from the analysis since this particular identifier is utilized by current databases that store information about regulatory relationships between genes. As a result, the first group contained 1860 downregulated genes and the second one – 1413 upregulated genes. The database GSEA [28] was used to search for regulatory elements influencing gene expression of these two groups. For every known today microRNA and TF, this resource allows to download a list of potential targets, that is genes having this microRNA's binding site or the recognition motif of TF respectively. Next, these gene lists can be compared to the groups of DE genes obtained earlier and through the application of appropriate statistical method, regulators enriched for these gene groups can be identified. In this context, regulators would be enriched for a gene group if they have statistically significant high number of targets among the genes of this particular group. The analysis just described was conducted for two groups of DE genes mentioned above.

For the group of downregulated genes (1860) 49 statistically significant (FDR < 0.05) enriched microRNA recognition sites were discovered (Supplementary Table 4), 36 of which corresponded to various microRNAs that had been linked to PCa earlier, such as miR-29 [29], miR-9 [30], miR-96 [31], miR-200 [32] etc. Association of 13 others with PCa was demonstrated in this study for the first time. These 3′-UTR motifs are recognized by the following microRNAs: miR-527, miR-374, miR-520F, miR-513, miR-142-5p, miR-507, miR-369-3p, miR-491, miR-511, miR-489, miR-432, miR-28, miR-520G and miR-520H. It is worth noting that every microRNA from this set had been associated with other oncological diseases including breast [33–36], ovarian [37, 38], colorectal cancers [39–41], glioblastoma [42, 43], non-small cell lung cancer [44, 45] and others.

Moreover, for the group of 1860 downregulated genes 340 statistically significant (FDR < 0.05) enriched TF binding sites were identified (Supplementary Table 5), 267 of which corresponded to known TFs and other 73 have not yet been attributed to any factor at the moment.

Many of the most represented motifs matched to recognition sites of serum response factor (SRF). This TF mediates signal from androgen receptor (AR) bypassing androgen-responsive elements (ARE). Furthermore, based on its target genes AR-SRF signature was determined that correlated with the presence of aggressive disease and poor outcome [46]. Expression of SRF gene, as well as that of its targets, was found to be lower in tumor tissue compared to control in this study (logFC = −0.8).

Another gene, encoding Myc-associated zinc-finger protein (MAZ) which interacts with AR, was also found at the top of enriched TF list. Its upregulation in tumor tissue in PCa had been discovered earlier and its knockdown by siRNA led to inhibition of cell proliferation and cell cycle arrest at G0/G1 phase [47]. In this study 353 of 3384 (10%) DE genes turned out to be its targets, all of them downregulated, and the expression of MAZ gene was found to be higher in tumor tissue compared to control which agrees well with the results of the previous studies. The list of enriched TF contained many other factors, whose role in PCa development had been demonstrated earlier: REPIN1 [48], NFAT [49], TCF3 [50], c-JUN [51], PAX4 [52] etc. In addition to these, we managed to identify 53 enriched TFs that had not been associated with PCa previously. Furthermore, 8 of them turned out tot be differentially expressed for the studied sample set: six (FOXJ2, GATA6, NFE2L1, NFIL3, PRRX2, TEF) downregulated and two (EBF2 and ZBTB18) upregulated in tumor tissue compared to control (Table 4). As in the case with microRNA, these transcription factors had been linked to other oncological diseases [53–59]. The fact that these factors suppress the expression of a significant number of genes in PCa and their own expression levels are altered makes them not only candidate biomarkers of prostate cancer but also potential therapeutic targets.

**Table 4:** Enriched TFs differentially expressed in this study

Gene symbol	Full name	LogFC	Number of downregulated genes, targeted by this TF
**EBF2**	early B-cell factor 2	3.1	53
**FOXJ2**	forkhead box J2	−0.5	71
**GATA6**	GATA binding protein 6	−1.7	57
**NFIL3**	nuclear factor, interleukin 3 regulated	−0.8	59
**PRRX2**	paired related homeobox 2	−1.9	37
**NFE2L1**	nuclear factor, erythroid 2 like 1	−0.4	74
**TEF**	TEF, PAR bZIP transcription factor	−1.1	42
**ZBTB18**	zinc finger and BTB domain containing	0.6	55

Interestingly, whereas the group of downregulated genes was highly enriched with TF and microRNA binding sites, not a single one of those was found to be enriched with an adequate level of statistical significance for the group of upregulated genes. Apparently, other mechanisms are involved in increasing expression of these genes in tumor tissue.

## MATERIALS AND METHODS

### Samples

Tissue samples were obtained from 26 patients with PCa and 4 patients with BPH from City Clinical Hospital No. 50 via radical prostatectomy (PCa) and transurethral resection of the prostate (TURP), respectively. All patients had not received specific therapy prior to sample collection. Information about smoking, kidney function, and concomitant drugs is provided in Supplementary Table 6 of supplementary material. The postoperative material was fixed in formalin and embedded in paraffin, the corresponding thin sections of FFPE tissue samples were examined by the pathologist who determined the areas of tumorous and histologically unchanged adjacent tissue.

### RNA extraction

AllPrep DNA/RNA FFPE Kit (Qiagen) и RNeasy FFPE kit (Qiagen) were used to extract RNA from FFPE samples according to manufacturer›s instructions. RNA quality was determined using 2100 Bioanalyzer (Agilent Genomics). DV_200_ values for all samples exceeded 63%. DNA contaminations were removed with the use of DNAse I (Fermentas) treatment following the manufacturer's recommendations. RNA concentration was determined by fluorometer Qubit 2.0 using Qubit RNA HS Assay Kit (Thermo Fisher). Ribosomal RNA depletion was performed on 200-1000 ng of total RNA by Low Input RiboMinus Eukaryote System v2 (Ambion).

### Transcriptome library preparation and sequencing

Transcriptome libraries were constructed using Ion Total RNA-Seq Kit v2 (Life Technologies) [60] with the following modifications of the protocol. For RNA fragmentation 1 ul of 10x RNase III buffer (Life Technologies) was added to 9 ul of RNA solution and heated for 10 min at 95°C followed by immediate snap-cooling on ice. After that 1 ul of 10 uM ATP and 1 ul of polynucleotide kinase (Fermentas) were added to the solution from the previous step and the whole mix was incubated at 37°C for 30 min. Fragmented RNA was cleaned up using Micro Bio-Spin Chromatography Columns (Bio-Rad). Further steps of library preparation including adapter ligation, first-strand cDNA synthesis and amplification were carried out in accordance with manufacturer's instructions. The prepared library was purified by magnetic beads Agencourt AMPure XP (Beckman Coulter Inc) and its quality was assessed by 2100 Bioanalyzer (Agilent Genomics) using Agilent High Sensitivity DNA Kit (Agilent Genomics). The sequencing of constructed transcriptome libraries was performed on Ion Proton platform using ION PI HI-Q Sequencing 200 Kit and Ion PI Chip Kit v2 (Life Technologies) following the recommendations of the manufacturer.

### Data analysis

The quality of reads was examined with the FastQC program [61] and their subsequent filtering and trimming were performed by Cutadapt [62]. Reads were then mapped to human genome version hg19 using STAR [63]. The HTSeq program [64] was used to count the number of reads mapped to a particular gene. The “htseq-count” python script was used with default parameters recommended by its author for most usages to yield a table where each gene Ensemble ID (Gencode Release 19 (GRCh37.p13) annotation) corresponded to a number of reads, overlapping this genomic feature. Reads corresponding to ribosomal RNAs were not included in further analysis. The comparison of the expression profiles, construction of the MDS plots and identification of differentially expressed genes were carried out using Bioconductor package edgeR [65]. Within this package the values obtained by HTSeq were normalized to library size, generating CPMs (counts per million reads). After exploring the data with the built-in MDS-plot function, removing outliers and defining the comparison groups differential expression analysis was performed based on Negative binomial model as explained in detail in the package documentation. The corresponding *p*-values were subjected to multiple-testing correction by Benjamini-Hochberg method [66]. Sequencing data has been deposited in the NCBI Gene Expression Omnibus (GEO) database under accession number GSE89223. The Cancer Genome Atlas (TCGA) data was downloaded as BAM files – results of RNA-Seq of 90 matched samples (IDs and clinical characteristics are provided in Supplementary Table 7). According to metadata of TCGA project, samples were derived from patients with primary prostate cancer that had not received any specific therapy. Based on BAM files raw sequencing data in the form of FASTQ files was obtained using Picard tools [67]. This, in turn, was subjected to the same pipeline as the Russian set using the same program set (described above in detail). A custom script was developed for discovering enriched binding sites of transcription factors and microRNAs. The statistical significance in this case was determined based on the hypergeometric distribution and multiple testing correction was performed by Benjamini-Hochberg method [66].

### RT-qPCR

For reverse transcription and qPCR 500 or 1000 ng of total RNA were used depending on the available quantity. DNA contaminations were removed with the use of rDNAse set (Machery-Nagel) following the manufacturer's recommendations. Half of each RNA sample was converted to cDNA with High-Capacity cDNA Reverse Transcription Kit (Applied Biosystems) according to the manufacturer's instructions. The other half was subjected to the same steps of cDNA conversion except for the addition of reverse transcriptase, thus providing negative controls for the further qPCR reaction. Real-time PCR was performed on CFX96 Touch Real-Time PCR Detection System (Bio-Rad) using qPCRmix-HS SYBR (Evrogen) following the manufacturer's recommendations. Sequencing data was used to choose the appropriate reference gene for qPCR normalization. Firstly, genes with low relative coefficients of variation were selected (this value can be calculated within the edgeR package). Then only the ones with high counts per million reads (CPM) value were considered as it indicated their high expression level in prostate tissue. Thus, Eukaryotic translation initiation factor 4 gamma 2 (EIF4G2) meeting all criteria was picked. This reference gene was used to normalize qPCR data for all genes under investigation. The sequences of primers used in this study are indicated in Table 5. For ANKRD34B gene the observed effect consisted in the absence of signal in control group samples vs. the presence of signal in tumor group, so the statistical significance was calculated using Ficher's exact test. For the rest of investigated genes corresponding deltaCt values were compared and *p*-values were derived from applying non-parametric Wilcoxon test.

**Table 5:** Primer sequences used for RT-qPCR

Primer ID	Sequence
ANKRD34Bf1	ACCCAAGCTGTCAACTGATCC
ANKRD34Br1	AGTCTTGTGAGGCGAAGCC
NEK5f1	GCCTTCGGGAAAGCATACTTAG
NEK5r1	AGGCTACAATGTTGGGATGTT
KCNG3f1	GGAGCAGGTACTCCGCCG
KCNG3r1	TACGGCGTGATTGCCAGTAA
PTPRTf1	TGGGAGAAACCAATGCTGGA
PTPRTr1	GCAGTGGGTGTCATTCTCCT
EIF4G2f1	ATTGTGGACAAAGCCCTAGAAG
EIF4G2r1	CTGGGCCATCAAAGTTTGGT

## SUPPLEMENTARY MATERIALS FIGURES AND TABLES













## Abbreviations

RNA-Seq: RNA sequencing
PCa: prostate cancer
BPH: benign prostatic hyperplasia
FFPE: formalin fixed paraffin embedded
MDS: multi-dimentional scaling
DE: differentially expressed
TCGA: The Cancer Genome Atlas
logFC: log2(Fold change)
TF: transcription factor.
