# Supplementary material for: Novel RNA biomarkers of prostate cancer revealed by RNA-seq analysis of formalin-fixed samples obtained from Russian patients
Source: Oncotarget. 2017 Mar 23;8(20):32990–3001. doi: 10.18632/oncotarget.16518 (PMC5464844; doi:10.18632/oncotarget.16518)
Supplement: Supplementary file 1 [file oncotarget-08-32990-s001.pdf]

Novel RNA biomarkers of prostate cancer revealed by RNA-seq analysis of formalin-fixed samples obtained from Russian patients

Supplementary Materials

Supplementary Table 1: Differentially expressed genes. See Supplementary_Table_1

Supplementary Table 2: Common DE genes for Russian and TCGA datasets. See Supplementary_Table_2

Supplementary Table 3: Samples used for qPCR validation

№	Patient ID	Diagnosis	Age at operation	PSA, ng/ml	Gleason score			Sample type used	
					Sum	Primary	Secondary	Pathology	Normal
1	A50_001	BPH	75	3.9	-	-	-	✓	✓
2	A50_002	BPH	67	1.5	-	-	-	✓	✓
3	A50_003	BPH	58	4.5	-	-	-	✓	✓
4	A50_006	PCa	64	17.0	7	4	3	-	✓
5	P50_005	PCa	67	11.0	7	3	4	✓	✓
6	P50_006	PCa	64	5.2	7	3	4	✓	✓
7	P50_012	PCa	68	14.9	7	3	4	✓	✓
8	P50_014	PCa	51	4.8	7	3	4	-	✓
9	P50_015	PCa	48	7.8	6	3	3	✓	✓
10	P50_016	PCa	67	6.6	5	3	2	✓	✓
11	P50_018	PCa	69	5.2	6	3	3	-	✓
12	P50_019	PCa	73	3.9	7	4	3	✓	✓
13	P50_020	PCa	50	12.0	7	3	4	✓	✓
14	P50_022	PCa	67	6.1	6	3	3	-	✓
15	P50_026	PCa	47	4.1	6	3	3	✓	✓
16	P50_027	PCa	61	5.9	7	4	3	✓	✓
17	P50_028	PCa	58	12.0	8	4	4	✓	✓
18	P50_036	PCa	40	7.0	7	3	4	✓	✓
19	P50_040	PCa	54	5.0	6	3	3	-	✓
20	P50_042	PCa	68	7.0	7	4	3	✓	✓

Clinical characteristics of the patients are indicated.

Supplementary Table 4: Enriched microRNA recognition sites for downregulated genes. See Supplementary_Table_4

Supplementary Table 5: Enriched TF binding sites for downregulated genes. See Supplementary_Table_5

Supplementary Table 6: Patient information about smoking, kidney function, and concomitant drugs

Patient ID	Smoking	Kidney function	Cardio drugs	Urological drugs
A50_001	no	normal	enalapril	silodosin
A50_002	no	no information	aspirin cardio	tamsulosin + dutasteride
A50_003	no	no information	losartan	tamsulosin + dutasteride
A50_004	no	no information	enalapril	tamsulosin + finasteride
A50_006	no	urea 39, creatinine 160	-	finasteride
P50_001	no information	no information	-	tamsulosin
P50_002	no information	no information	-	-
P50_004	no information	no information	-	silodosin
P50_005	no information	no information	-	silodosin
P50_006	yes	normal	-	-
P50_007	no	urea 26, creatinine 149	-	-
P50_008	yes	normal	enalapril	tamsulosin
P50_009	no	normal	-	tamsulosin
P50_010	no	no information	metoprolol	-
P50_011	no information	normal	losartan	tamsulosin + finasteride
P50_012	no information	no information	enalapril	-
P50_013	yes	normal	-	-
P50_014	no	urea 20.5 creatinine 130	rivaroxaban	tamsulosin + dutasteride
P50_015	yes	normal	-	-
P50_016	no	no information	enalapril	finasteride
P50_018	no	normal	rivaroxaban	silodosin
P50_019	yes	urea 19, creatinine 151	losartan	-
P50_020	yes	normal	-	tamsulosin
P50_022	yes	normal	aspirin cardio	-
P50_026	no information	normal	-	-
P50_027	no	urea 15, creatinine 140	losartan	silodosin
P50_028	no	normal	-	-
P50_036	no	no information	-	finasteride
P50_040	yes	normal	-	-
P50_042	yes	normal	-	tamsulosin

Supplementary Table 7: Sample information for TCGA dataset. See Supplementary_Table_7
